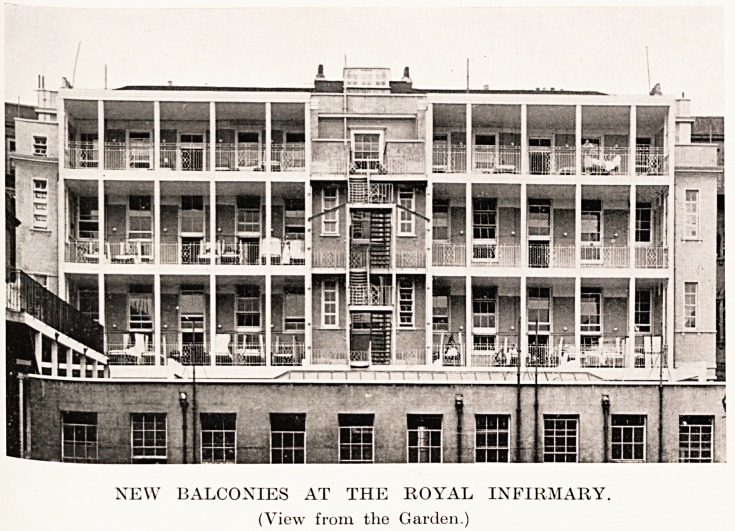# Editorial Notes

**Published:** 1936

**Authors:** 


### Editorial Notes

**The Royal Medical Benevolent Fund Centenary.**

THE Council of this Fund is making a special appeal for new subscribers the main feature of the centenary. It points out that during the hundred years since the foundation the Fund has distributed nearly £400,000 to medical practitioners in difficulties due to sickness or infirmities, and to their widows and families left without provision. It is particularly desired to increase allowances to disabled practitioners to £52, and to their dependants to £36 per annum: and to create a special fund for the relief of very urgent and distressing cases.

The fact that at present allowances have necessarily to be substantially less than these very small sums speaks eloquently of the real need of the beneficiaries. But only those who have had personal experience of administration can fully appreciate how sad is the position of a professional man or his widow in such poverty that a grant so small as this means salvation from the workhouse—as too often it does.

The Fund is a charity: that is to say, it does not matter whether the beneficiary has in happier times been a subscriber or not. And it is a charity supported entirely, as is right, by contributions from the medical profession, every member of which should regard it as a duty, no less than a privilege, to subscribe regularly and generously. Subscriptions should be sent to the Treasurer, at 11 Chandos Street, London, W.1.

**B.R.I. New Sisters' Hostel and Balconies.**

THE following account is taken from the report which appeared at the time in the *Western Daily Press.*

"Tucker House," the new sisters' hostel at the Bristol Royal Infirmary, was officially opened on Friday, July 17th, by the Duchess of Beaufort.

The President (Lieut.-Colonel P. G. Robinson), introducing the Duchess, said that the committee had decided to call the new hostel "Tucker House," in recognition of a large legacy bequeathed to the institution by the late Mr. C. H. Tucker. The architect was Mr. H. Hill, the clerk of works, and the work was done by direct labour at a cost of £2,100. The building would accommodate twelve sisters.

Mr. Hill handed a key to the Duchess, who then unlocked the door and declared the hostel open.

After inspecting "Tucker House" the Duchess crossed over to the old block and declared open the new balconies which have been erected on the southern aspect of the wards. She was handed scissors and cut a ribbon across the staircase in declaring the balconies open. The President explained that Mr. Hill was also the architect of the balconies and that the work in this case was also done by direct labour at a cost of £3,250. The balconies provide accommodation for thirty-six to forty patients. A Garden Party and Nurses' Reunion were later held, although the rain interfered to some extent with the proceedings.

The old building of the Infirmary won a remarkable tribute from a Spanish visitor in 1935, who said she had visited hospitals and clinics all over the world and she had thought that any hospital building that

PLATE XVIII

NEW BALCONIES AT THE ROYAL INFIRMARY.
(View from the Garden.)

was 200 years old could only be pulled down. The modern adaptation of the Bristol Royal Infirmary had taught her a lesson she would never forget.

**Joseph Rogers Prize.**

FOR the second time since its institution the Joseph Rogers Prize has been allotted to a Bristolian, and we congratulate Dr. Llewelyn Roberts, Assistant Medical Officer of Health, on his success. Ten years ago, when the first award was made, the successful essayist was H. J. McCurrich, M.S., F.R.C.S., who formerly lived in Clifton, was educated at Clifton College, and studied medicine for a time in Bristol University.

The prize is provided by a bequest of the late Dr. Joseph Rogers, a distinguished pioneer in public health work, who at his death in 1889 left a sum of £500 for the purpose of providing a prize every ten years "for such person who, in the opinion of the trustees, shall write the best or only good essay on the treatment of the sick poor, or the preservation of the health of the poor of this country, or on either of these subjects."

The trustees nominated by Joseph Rogers for the administration of this bequest were the Master of the Worshipful Society of Apothecaries and the President of the Royal College of Physicians. The prize has only been awarded once before, and amounts to between £200 and £300.

Dr. Joseph Rogers was brother of the late Professor Thorold Rogers, and uncle of Dr. Bertram Rogers, Consulting Physician to the Royal Hospital for Sick Women and Children in Bristol, and formerly Regional Medical Officer to the Ministry of Health.

**Visit of Minister of Health to Bristol.**

THE Minister of Health, Sir Kingsley Wood, P.C., M.P., visited Bristol on 16th September, 1936, spending two days in inspecting the various health services and housing conditions.

On his arrival, the Minister referred to the Portway Joint Clinic, where an arrangement has been made for a Health Visitor to carry out routine inspections at the schools in the area. He said that the scheme adopted at this Clinic was unique, and was being carefully studied at the Ministry. Such close co-operation between the Education and Health Committees in the interests of the children gave him great satisfaction.

The Minister visited Ham Green and Southmead Hospitals, and on the afternoon of 17th September he opened a new school and nursery centre at Ilminster Avenue. The Nursery Centre is administered on the lines of a nursery school, but is under the control of the public health authority in place of the Education Committee. It will form part of a training school for nursery nurses with its base at the Downend Babies' Home.

At the Preventive Medicine Department (Canynge Hall) the Minister commended the close relationship between the public analyst and the other sections in the interest of supervision of food supplies, a matter, as he said, of extreme importance to the vast numbers of people who live in this area.

In a speech at the Mansion House in the evening the Minister praised the health services of the city. He referred to the low infant mortality and maternal mortality rates, to the improved housing, and to the clinics and hospitals. He was satisfied that Bristol was maintaining a good standard of efficiency and progress in the discharge of its responsibilities. He also paid a tribute to the work of the Corporation in slum clearance and rehousing: the establishment of a Community Centre at Knowle West housing estate won his particular admiration.

He expressed satisfaction at the improved health of the school children, who were healthier, taller and heavier than their predecessors. The Minister also spoke of the diseases that had disappeared, but whilst much had already been done, he reminded his audience of what remained to be done. He wished to stimulate action in regard to tuberculosis, cancer, respiratory and mental diseases.

Finally, he said that the health of our people at large is by no means satisfactory. To sum it up in a few short words, we must have a more and more positive policy in the matter of health services, aiming not merely at safeguards against disease, but at the active promotion of the health of the people.

**Extensions at Winford Orthopædic Hospital.**

ON Saturday, 10th October, Lord Kennet (who, as Sir Hilton Young, was Minister of Health) opened two new Wards and an isolation block at Winford Orthopædic Hospital, which will increase the total beds by 40, making a total of 108. This extension has been, in part, financed by the shareholders of Bristol Housing Ltd., a company formed in 1909 for the purpose of developing a garden suburb at Shirehampton (Vol. LI., No. 194, page 279). This company had accumulated a reserve fund of £10,000. In 1934 it was decided to wind up the company: the shareholders' capital was returned, but as they had resolved to make no profit out of the estate at Shirehampton the reserve fund was given to Winford Orthopædic Hospital to build new Wards. In addition to the new Wards the accommodation at the Nurses' Home has been increased. The total cost of the extensions is nearly £20,000.

### Meetings of Societies

## Figures and Tables

**Figure f1:**